# A lower dose of intravitreal conbercept effectively treats retinopathy of prematurity

**DOI:** 10.1038/s41598-018-28987-6

**Published:** 2018-07-16

**Authors:** Yong Cheng, Qingyu Meng, Dandan Linghu, Mingwei Zhao, Jianhong Liang

**Affiliations:** 1 Department of Ophthalmology, Peking University People’s Hospital, Beijing, China; 2 Eye diseases and Optometry Institute, Beijing, China; 3 Beijing Key Laboratory of Diagnosis and Therapy of Retinal and Choroid Diseases, Beijing, China; 4 College of Optometry, Peking University Health science center, Beijing, China

## Abstract

Intravitreal Conbercept (IVC) is the latest applied and effective treatment for the management of retinopathy of prematurity (ROP). However, conbercept escapes from the vitreous into the general circulation and reduce systemic VEGF concentrations. Thus, there are concerns about systemic complications, in these premature infants who are developing vital organ systems. This study is to determine whether a low dosage (0.15 mg/0.015 mL) of IVC is effective in the treatment of Zone II Stage 2/3 + ROP. A total of 38 eyes of 20 infants were analyzed retrospectively. We identified treatment effectiveness as complete regression of retinopathy and retinal vascularisation to zone III. The mean gestational age (GA), postmenstrual age (PMA) at treatment and birth weights (BW) were 28.6 ± 2.2 weeks, 39.3 ± 3.0 weeks and 1297.5 ± 429.2 g respectively. Primary effectiveness (react to IVC 0.15 mg alone) was found in 32/38 eyes (84.2%). Secondary effectiveness (a second IVC was required) was found in 6/38 eyes (15.8%). Follow-up continued until 90 weeks’ postmenstrual age and showed no recurrences of plus disease or neovascularization. The study suggests 0.15 mg IVC is effective for Zone II Stage 2/3 + ROP, and there is no adverse ocular outcomes during the follow-up period.

## Introduction

Retinopathy of prematurity (ROP) is a neovascular disorder that occurs in premature infants and is the leading cause of infant blindness, especially in developing countries due to inappropriate neonatal care and a lack of local ROP examination programs^1–4^. Vascular endothelial growth factor (VEGF) is believed to play an important role in the pathogenesis of ROP^5–7^. Laser has been the gold standard treatment for type 1 ROP of all forms of ROP, which can reduce the overproduction of VEGF and induce the regression of neovascularization by ablating peripheral avascular retina. However, this therapy may cause complications, such as peripheral visual field defect and myopic shift. Moreover, laser therapy is difficult to undertake when there is poor visualization of the retina from vitreous haze or inadequate pupil dilatation in severe ROP^3,8,9^.

Recently, anti-VEGF treatment with bevacizumab has been shown to be effective in treating ROP in the BEATROP trial^2^, which showed that vascularization was completed 19.5 weeks after intravitreal bevacizumab (IVB). The rate of recurrence with zone I disease was 6% (2 of 31 infants) with intravitreal bevacizumab and with zone II posterior disease was 5% (2 of 39 infants). Jiang *et al*. showed 39.0% (245of 629 eyes) recurrence rate after intravitreal ranibizumab (IVR) with the reappearance of neovascularization and the return of plus disease in China^10^. Menke *et al*. reported that, after IVR, vascularization of the retina was completed between the 3rd and 6th months without recurrence^11^. Jin *et al*. showed 85% (17 of 20 eyes) regression of ROP after receiving the injection conbercept (IVC) only once, and the regression of plus disease occurred 4.3 ± 2.08 weeks later^12^.

However, not only bevacizumab but also ranibizumab escape from the vitreous into the general circulation and reduce systemic VEGF concentrations for weeks to months^13,14^. Thus, there are concerns about systemic complications, in these premature infants who are developing vital organ systems. Considering the potential adverse effects of suppressing VEGF levels in both local and systemic environments in premature infants, lower dosage of conbercept in the ROP eye would be desirable, if equivalent efficacy could be achieved. The usual dosage for conbercept is 0.25 mg/0.025 ml for ROP. In this study, we investigated whether a lower conbercept dosage (0.15 mg/0.015 ml) is effective in the treatment of stage 2+ or 3+ ROP in Zone II posterior disease.

## Methods

The study was a retrospective study approved by the Institutional Review Board of Peking University People’s Hospital (Beijing, China), which was conducted in adherence to the tenets set forth in the Declaration of Helsinki. Written informed consent was obtained from the parents of each infant before receiving the intravitreal injection of Conbercept. Before treatment, we discussed with the parents of all affected children the possibility of using off-label IVC as an alternative to conventional standard laser treatment performed according to the international guidelines of the ROP^1,2,15^.

The infants who visited People’s Hospital of Peking University in Beijing, China between August 2016 and November 2016 were included in the study and were followed-up until 90 weeks postmenstrual age. Our study included thirty-eight children who were consecutively treated for stage 2+ or 3+ ROP disease in zone II. Examination of binocular indirect ophthalmoscopy taken by two experienced ophthalmologists (Y.C. and J.H.L.) was important for treatment decision. The definitions of stage and zone were based on the revised guidelines of the International Committee for the Classification of Retinopathy of Prematurity^1^. Infants with any eye disease other than ROP, such as glaucoma and congenital cataract, and infants with a history of prior treatment for ROP were excluded.

Our procedure of intravitreal injection was performed according to guidelines of an expert panel^16^. In each case, 0.015 mL (0.15 mg) of the drug was administered via a 300 µL syringes with 5/16-inch, 30-gauge fixed needles, 1.5 mm posterior to the limbus. Infants were reexamined at day 1 after treatment and then examined every twice a week until the ROP completely regressed. The next follow-up visits were held weekly for four weeks, biweekly for the next eight weeks, and then monthly until 90 weeks postmenstrual age. RetCam photos (Clarity Medical Systems, Inc., Pleasanton, CA) were taken before and after treatment for each follow-up.

We defined the primary outcome studied as structural outcomes, including regression of plus disease, the disappearance or decrease of retinal vessel tortuosity and neovascularization, and the growth of the normal retinal vessels toward the peripheral retina. Secondary outcome was defined as the presence of recurrence, times of injection, and the final regression of the disease. The recurrence of ROP was defined as the recurrence of retinal abnormality, such as ridge and plus disease after their regression. In the case of early treatment failure, a second IVC was performed. If persistent or progressive activity (progressive plus disease, fibrovascular proliferation and vascular changes at the junction vascular/avascular retina) after a second IVC was shown, it was treated with laser therapy, and vitrectomy for retinal detachment. Major complications were defined as corneal opacity requiring corneal transplant, lens opacity requiring cataract surgery, preretinal or intravitreal hemorrhage requiring vitrectomy, and tractional retinal detachment after IVC.

Descriptive continuous variables were presented using the mean and standard deviation and and the Mann-Whitney U test was used to test the differences between groups. Categorical variables were reported as proportions (%) and compared between the groups using χ^2^ test and Fisher’s exact test. The potential clinical factors were included in univariate analysis. Statisticalanalyses were performed using SPSS 22.0 for Windows (SPSS,Inc., Chicago, IL, USA). A P < 0.05 (2-sided) was considered significant for all tests.

## Results

The study included 38 eyes of 20 infants. The mean gestational age (GA) was 28.6 ± 2.2 weeks (mean ± standard deviation; range, 24–33 weeks), and birth weights (BW) was 1297.5 ± 429.2 g (mean ± standard deviation; range, 800–2300 g) respectively. The mean postmenstrual age (PMA) was 39.3 ± 3.0 weeks (mean ± standard deviation; range, 34–44 weeks) (Table 1). Among 20 infants, eight were male (40%), and twelve were female (60%). All children showed stage 2+ or 3+ ROP diseases, in zone II. Among 38 eyes, 10 eyes were zone II stage 2+ and eighteen eyes were stage 3+.

**Table 1:** Baseline demographics.

Patient	GA(weeks + days)	BW(g)	Eye	Zone	Stage	‘Plus’	PMA at treatment (weeks + days)	Dosage(mg)	PMA at latest follow-up (weeks)	Need for retreatment	Response to treatment
1	32 + 1	1650	OD	2	2	+	36 + 3	0.15	98	NO	Primary effectiveness
			OS	2	2	+		0.15		NO	Primary effectiveness
2	29 + 4	1500	OD	2	2	+	34 + 1	0.15	132	YES	Secondary effectiveness
			OS	2	2	+		0.15		YES	Secondary effectiveness
3	25 + 3	930	OD	2	3	+	40 + 2	0.15	94	NO	Primary effectiveness
			OS	2	3	+		0.15		NO	Primary effectiveness
4	26 + 6	900	OD	2	3	+	36 + 4	0.15	92	NO	Primary effectiveness
			OS	2	3	+		0.15		NO	Primary effectiveness
5	31 + 2	1150	OD	2	3	+	41 + 1	0.15	96	NO	Primary effectiveness
			OS	2	3	+		0.15		NO	Primary effectiveness
6	28	1050	OS	2	3	+	36	0.15	92	NO	Primary effectiveness
7	24	950	OD	2	3	+	37	0.15	92	NO	Primary effectiveness
			OS	2	3	+		0.15		NO	Primary effectiveness
8	28	2140	OD	2	3	+	42 + 2	0.15	90	NO	Primary effectiveness
			OS	2	3	+		0.15		NO	Primary effectiveness
9	27	800	OD	2	3	+	37 + 4	0.15	102	NO	Primary effectiveness
			OS	2	3	+		0.15		NO	Primary effectiveness
10	29 + 4	2300	OS	2	3	+	38 + 6	0.15	98	NO	Primary effectiveness
11	27 + 6	1080	OD	2	2	+	42 + 6	0.15	104	NO	Primary effectiveness
			OS	2	2	+		0.15		NO	Primary effectiveness
12	27 + 6	1080	OD	2	2	+	42 + 6	0.15	104	NO	Primary effectiveness
			OS	2	2	+		0.15		NO	Primary effectiveness
13	28 + 5	1500	OD	2	3	+	35 + 5	0.15	96	YES	Secondary effectiveness
			OS	2	3	+		0.15		YES	Secondary effectiveness
14	28 + 6	1150	OD	2	3	+	44 + 3	0.15	99	NO	Primary effectiveness
			OS	2	3	+		0.15		NO	Primary effectiveness
15	27 + 1	1100	OD	2	3	+	38	0.15	99	NO	Primary effectiveness
			OS	2	3	+		0.15		NO	Primary effectiveness
16	27	970	OD	2	3	+	44	0.15	99	NO	Primary effectiveness
			OS	2	3	+		0.15		NO	Primary effectiveness
17	33	1400	OD	2	2	+	38	0.15	144	YES	Secondary effectiveness
			OS	2	2	+		0.15		YES	Secondary effectiveness
18	29 + 2	1500	OD	2	3	+	41 + 1	0.15	99	NO	Primary effectiveness
			OS	2	3	+		0.15		NO	Primary effectiveness
19	27 + 6	900	OD	2	3	+	39 + 6	0.15	94	NO	Primary effectiveness
			OS	2	3	+		0.15		NO	Primary effectiveness
20	31 + 5	1900	OD	2	3	+	39 + 4	0.15	98	NO	Primary effectiveness
			OS	2	3	+		0.15		NO	Primary effectiveness

BW, birth weight; GA, gestational age; PMA, postmenstrual age; OD, right eye; OS left eye.

**Figure 1:**
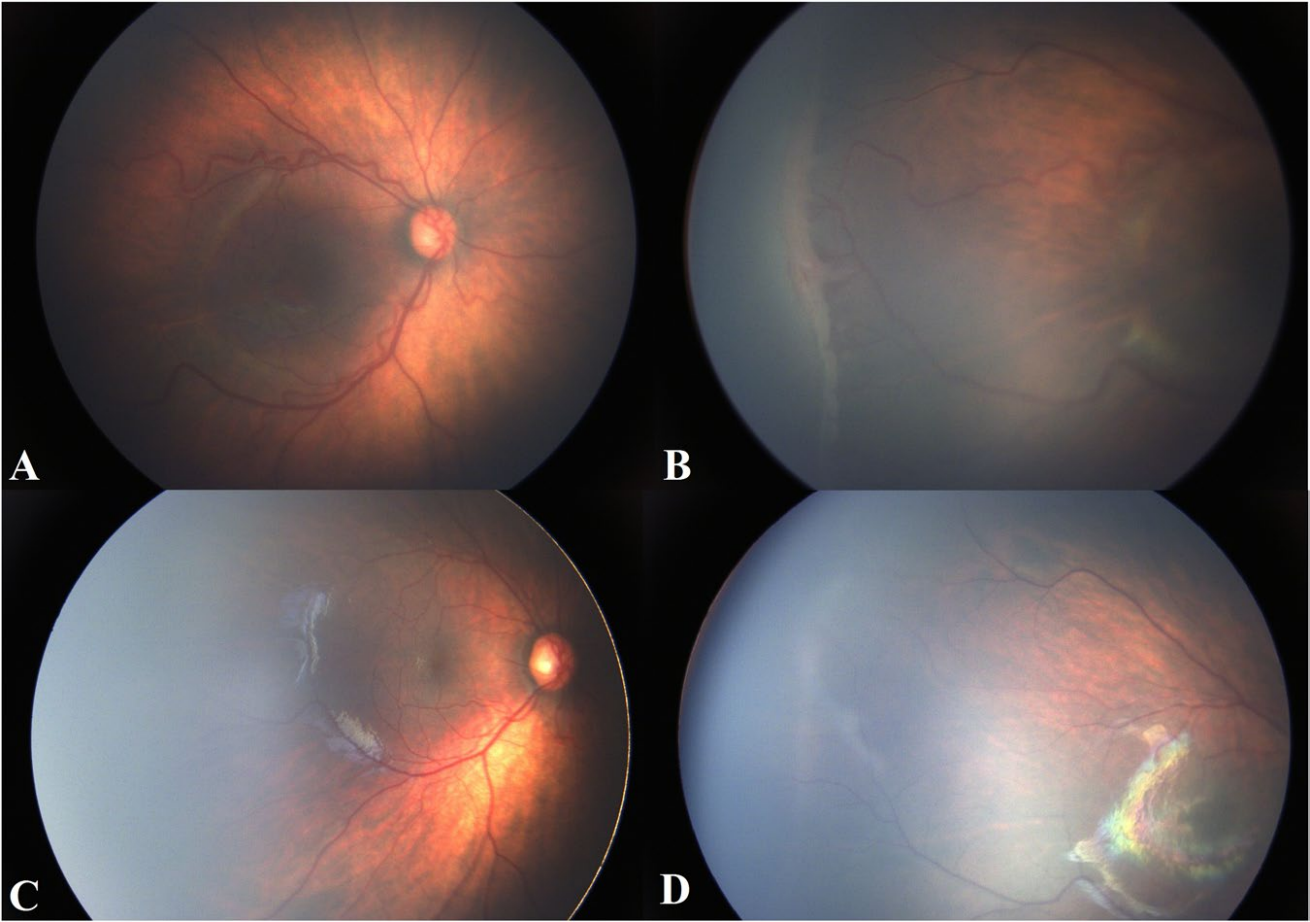
Patient 16, Male, GA 27 weeks, BW 970 g, treated at 44 weeks’ PMA. He had stage 3+ in zone II ROP diseases, received only one injection of conbercept after the first fundus examination, and the situation of plus disease and ridge was consecutively observed for 55 weeks. (**A**,**B**) Shows the right eye with plus and ridge before conbercept injection. (**C**,**D**) Shows the same eye regression of plus disease, ridge and the disappearance or decrease of retinal vessel tortuosity, and the growth of the normal retinal vessels toward the peripheral retina 55 weeks after conbercept injection.

**Figure 2:**
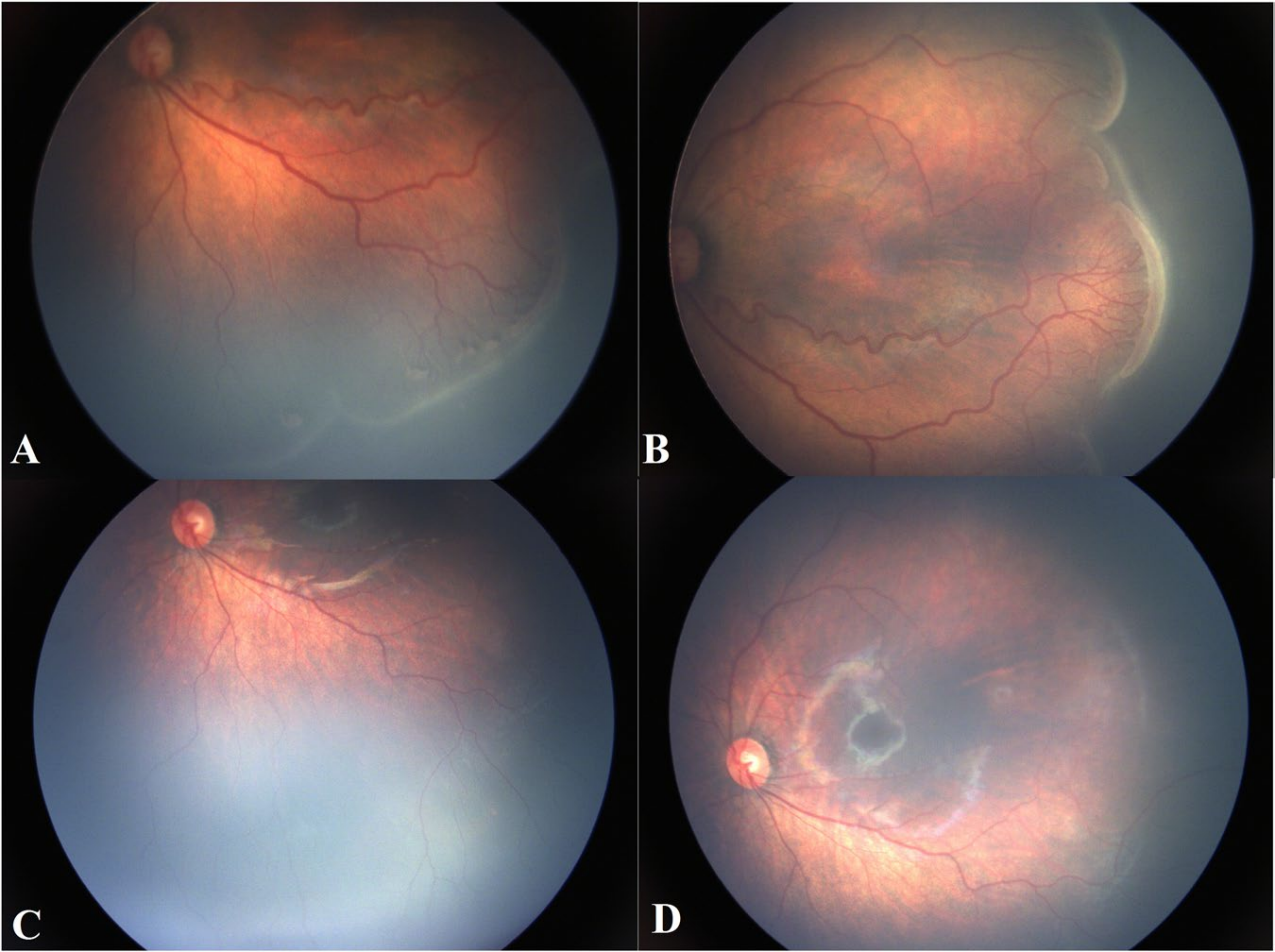
Patient 7, Female, GA 24 weeks, BW 950 g, treated at 37 weeks’ PMA. She had stage 3+ in zone II ROP diseases, received only one injection of conbercept after the first fundus examination, and the situation of plus disease and ridge was consecutively observed for 55 weeks. (**A**,**B**) Shows the left eye with plus and ridge before conbercept injection. (**C**,**D**) Shows the same eye regression of plus disease, ridge and the disappearance or decrease of retinal vessel tortuosity, and the growth of the normal retinal vessels toward the peripheral retina 55 weeks after conbercept injection.

The final follow-up visit of infants were for a mean PMA of 101.1 weeks (range 90–144 weeks). We identified treatment effectiveness as complete regression of retinopathy and retinal vascularisation to zone III. Primary effectiveness (react to IVC 0.15 mg alone) was found in 32/38 eyes (84.2%)(Figs 1 and 2). Secondary effectiveness (a second IVC required) was found in 6/38 eyes (15.8%). Additional a second IVC was implemented at a mean of 9.29 weeks after first IVC, and at a mean of 44 weeks’ PMA (range 35–38). No systemic or ocular complications or side effects were observed during the follow-up period.

## Discussion

Since the BEAT-ROP trial, intravitreal anti-VEGF for ROP has gained broad popularity in clinics^2,12,15,17^. The present “standard” dosage of IVC for ROP (0.25 mg in 0.025 mL) stands for a origin of adult doseage of Conbercept treated for conditions such as AMD and retinal vein occlusion (0.5 mg in 0.05 mL)^18,19^. The dosage used in the treatment of ROP^12^, 0.25 mg, is half the adult dosage. However, the vitreous volume of an infant at 34 weeks is less (1.6 ml vs 4.0 ml), as is the retinal surface area (450 mm^2^ vs 1240 mm^2^), and the body weight is about one-fiftieth of an adult, Nonetheless, found a relatively high dosage of Conbercept was given.

As we know, after intravitreal anti-VEGF injection, anti-VEGF enters the systemic circulation in animal models and humans, lowering the serum level of VEGF. Following an intravitreal injection of 1.25 mg into adult macaques, one eye gave peak serum concentrations of the antibody at one week and was still detected at 8 weeks^20^. There are similar data in newborn rabbits with higher serum concentrations, which were shown when the intravitreal injection was given in the earlier neonatal period^21,22^. Moreover, Wu found serum VEGF levels were suppressed for 2 months after IVB in patients with type 1 ROP, owing to the leakage of bevacizumab into the systemic circulation, and Hoerster showed ranibizumab suppressed systemic VEGF levels below detection limits for approximately 2 weeks, reaching a nadir at 2-3 weeks after intravitreal injection^14,23^. However, VEGF plays an important role in the development of most neural tissues and organs, particularly in newborns. Preterm infants are still undergoing organogenesis at the time of ROP treatment late in the third trimester. In the eye, VEGF is required for normal neural retinal development independent of angiogenesis^7^. In the lungs, the pneumotrophic effect of VEGF may have therapeutic potential for lung maturation in preterm infants^5^. It is now apparent that VEGF induces neuritic growth and provides neuroprotection, particularly after ischemia or spinal cord injuries, and has an additional role linking the coordinated patterning of developing vascular and nervous tissue in the brain^6^. Based on the dosage relationship between the vitreous concentration of anti-VEGF and the serum concentration of anti-VEGF, the lowest possible dosage of anti-VEGF should be recommended for infants with ROP.

Many studies have showed it is effective as a lower dosage to anti-VEGF for ROP. Khodabande *et al*. showed 49 eyes with type 1 ROP treated with 0.25 mg/0.01 mL of IVB, and there was regression of plus disease/extraretinal neovascularization in all eyes during the first weeks after treatment and no recurrence of ROP in any eyes by 90 weeks gestation^24^. Hillier *et al*. have reported more favorable outcomes for IVB treated with 0.16 mg^25^. Moreover, Wallace DK found that a dosage of bevacizumab as low as 0.031 mg, 5%of the dosage used in the BEAT-ROP trial, was effective in 9 of 9 eyes^26^. Recently, Ells *et al*. reported forty-two eyes of 21 infants with type 1 ROP received a 0.2 mg (0.02 mL) intravitreal injection of ranibizumab as the primary treatment. Active neovascularization regressed rapidly, and anatomical outcomes were favorable in all eyes^27^. Our team previously showed series of ROP regression to standard dosage Conbercept 0.25 mg^12^. In this paper, we first reported a series of ROP infants treated with lower dosage Conbercept.

We have previously shown that, regression of plus disease, the disappearance or decrease of retinal vessel tortuosity and neovascularization may be expected within 48 hours. Additional treatment rate in our cases was 15.8% (eyes), and all children who received a second IVC finally representing well involution. Still, the rate of retreatment appeared a little higher than that represented recently. At our previous study reported a second IVC rate of 15% (17/20) in children treated as 0.25 mg IVC^12^. Because of different drug dosages, it is difficult to make comparisons. The mean BW and GA for the infants were 1,297 ± 429 g and 28.56 ± 2.24 weeks, respectively, in our cohort, and 29.49 ± 1.37 weeks and 1,369.0 ± 161.9 g, respectively, in Jin’s cohort. It may appear that we have a higher GA and BW. Moreover, we found that the need for retreatment arose at 9.29 weeks post-IVC in our study and at 4.12 weeks in Jin’s report. It seems logical to conclude that the lower dosage IVC may show a different lengthen of effective drug concentration from the standards, and it is likely that the recurrence take place more swiftly. However, our results showed that the time of retreatment that arose with the lower dosage was later than that with standard. It is also possible that the subjects of our study were all stage 2 + or 3 + ROP disease in zone II, which was milder than those in zone I or AP-ROP.

This study was limited by the retrospective design, and the results should be confirmed with a prospective study and a large patient enrollment. Nonetheless, our outcome supports the efficacy of a lower dosage of IVC for ROP in some infants.
